# Specific pathogens and microbial abundance within liver and kidney tissues of wild marine fish from the Eastern Mediterranean Sea

**DOI:** 10.1111/1751-7915.13537

**Published:** 2020-02-14

**Authors:** Dalit Meron, Nadav Davidovich, Maya Ofek‐Lalzar, Ran Berzak, Aviad Scheinin, Yael Regev, Rei Diga, Dan Tchernov, Danny Morick

**Affiliations:** ^1^ Morris Kahn Marine Research Station Department of Marine Biology Leon H. Charney School of Marine Sciences University of Haifa Haifa Israel; ^2^ Israeli Veterinary Services Bet Dagan Israel; ^3^ Bioinformatics Services Unit University of Haifa Haifa Israel

## Abstract

This study is an initial description and discussion of the kidney and liver microbial communities of five common fish species sampled from four sites along the Eastern Mediterranean Sea shoreline. The goals of the present study were to establish a baseline dataset of microbial communities associated with the tissues of wild marine fish, in order to examine species‐specific microbial characteristics and to screen for candidate pathogens. This issue is especially relevant due to the development of mariculture farms and the possible transmission of pathogens from wild to farmed fish and vice versa. Although fish were apparently healthy, 16S rRNA NGS screening identified three potential fish bacterial pathogens: *Photobacterium damselae, Vibrio harveyi* and *Streptococcus iniae*. Based on the distribution patterns and relative abundance, 16 samples were classified as potential pathogenic bacteria‐infected samples (PPBIS). Hence, PPBIS prevalence was significantly higher in kidneys than in liver samples and variation was found between the fish species. Significant differences were observed between fish species, organs and sites, indicating the importance of the environmental conditions on the fish microbiome. We applied a consistent sampling and analytical method for monitoring in long‐term surveys which may be incorporated within other marine fish pathogens surveys around the world.

## Graphical abstract

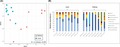

## Introduction

**Figure 1:**
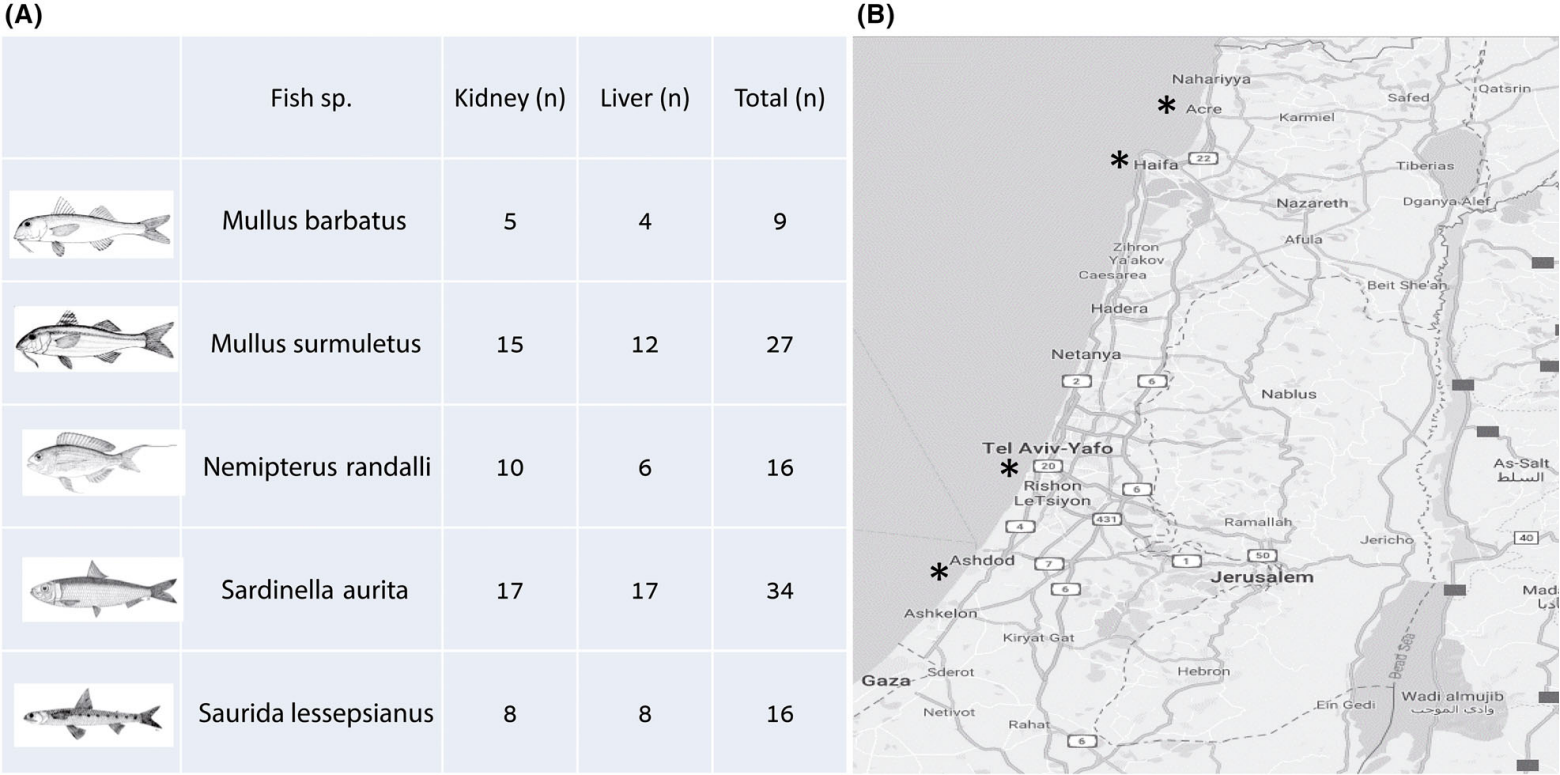
Fish species and sampling sites. Summarizing table of fish species and number of samples (kidney and liver). Fish illustrations were taken from FAO (Food and Agriculture Organization of the United Nations) site, http://www.fao.org/fishery/en (A). Israeli shoreline map including the four sampling sites (Acre, Kishon (near Haifa), Jaffa and Ashdod) marked with asterisks (B).

In recent years, there is a growing interest in the marine holobiont concept (Cahill, 1990; Apprill, 2017) and efforts are being made to collect data regarding the microbial communities of various marine organisms, including invertebrates such as corals (Weber *et al.*, 2017), sponges (Webster *et al.*, 2010), hydra (Deines and Bosch, 2016), higher vertebrates such as fish (Egerton *et al.*, 2018) and marine mammals (Apprill *et al.*, 2014). Describing the host microbiome enables us to define the specific microbial communities of each organism, to trace changes in these communities, and to identify potential pathogens. Changes within host microbiota may be due to environmental changes (Cahill, 1990), anthropogenic impacts (Halpern *et al.*, 2008) and/or host physiology (Clements *et al.*, 2014); hence, the microbiome characterization may be used as an important bio‐indicator to assess the host's health status and the marine environment's microbial abundance over time and place. The microbial community structure is very dynamic and subject to rapid shifts; therefore, changes in its composition and the modulation of pathogens prevalence may indicate changes in environmental condition even before their appearance. Microbiome balance is known to be key for maintaining overall health status in fish (Gómez and Balcázar, 2008), as shifts in the microbiome in response to stressors could be a precursor to disease, and thus are of crucial practical importance in aquaculture and pathogen monitoring surveys. Multiple phenomena could be potentially addressed through elucidation of the microbial community, including nutrient digestion, synthesis and absorption, pathogen resistance, growth, sexual maturation, morphogenesis, stock survivorship and more (Llewellyn *et al.*, 2014). Prior studies have focused on the skin, gill, mucosa and mainly intestinal content microbiome (Larsen *et al.*, 2013; Llewellyn *et al.*, 2014; Tarnecki *et al.*, 2017), while other organs such as kidney, liver or spleen are underrepresented; only a few studies have been conducted on wild marine species’ internal tissues microbiomes (Sevellec *et al.*, 2014; Türe and Alp, 2016). Two important internal organs were chosen in our study for microbiome characterization: the liver, which is the central metabolic organ and has a significant importance in the maintenance of overall nutrition and homeostasis of fish (Hampton *et al.*, 1985, 1988, 1989) including immune responses (Morales *et al.*, 2004; Martin *et al.*, 2010) and the kidney, which has plays several functional roles, including osmoregulation and immune functions (Calderwood, 1891; Hickman and Trump, 1969; Tort *et al.*, 2003). The internal organs, in contrast to gills and skin, do not directly contact the outer environment, and thus may represent the organ’s specific symbiotic microbiome and provide important information. Already 30 years ago, a wide range of bacteria in kidney and liver of fish (Mudarris and Austin, 1988; Toranzo *et al.*, 1993; Starliper and Tesk, 1995) was reported and in recent years more studies have focused on the presence of bacteria in apparently healthy fish internal organs (Pujalte *et al.*, 2003; Suzuki, 2018). One explanation for the presence of bacteria in what should be considered a ‘sterile organ’ is the breakdown of immunological defence mechanisms as a result of stress, which enhances the presence of bacteria in immune system cells and in the blood’s circulation (Tort, 2011). Stress factors include poor water quality, temperature changes, nutritional deficiencies, overcrowding, trauma and infection (Americo De Sousa *et al.*, 1999), which all exist in variable levels within the marine environment. The World Organization for Animal Health (OIE) recommends sampling of apparently healthy fish internal organs for the most reported diseases (OIE, 2001). In addition, most fish studies were conducted on mariculture fish with high economic value (OIE, 2001; Martínez‐Porchas and Vargas‐Albores, 2017) and from freshwater habitats (Tarnecki *et al.*, 2017) while, there is lack of basic information about marine wild fish microbiome and pathogens (Ward and Lafferty, 2004; Arechavala‐Lopez *et al.*, 2013), even though they are the most diverse group of vertebrates in the marine environment. Marine fish occupy a wide range of habitats, exhibit high‐variety diet composition, and often play important trophic and ecological roles. Our survey was done along the continental shelf of the Eastern Mediterranean Sea, along the Israeli shoreline, which is an area under unique environmental conditions and exposed to anthropogenic influences. The Mediterranean Sea is seasonally biodiverse due to its variable geophysical and biogeographical properties in its different basins, and this unique position might provide several insights into larger‐scale marine ecosystems (Lejeusne *et al.*, 2010). Monitoring the marine environments’ water and sediment column, as well as different marine organisms, enables us to examine each site’s condition and to observe further changes following environmental changes and/or anthropogenic impacts. As part of an annual monitoring survey of fish pathogens along the Israeli shoreline, we focused on five representative fish species (Fig. 1) which are common in the area, and we characterized their liver and kidney microbial communities. The goals of the present study were as follows: (i) to establish a baseline dataset of the microbial communities associated with the kidney and liver tissues of wild common marine fish; (ii) to examine species‐specific microbiome characteristics; (iii) to examine species‐specific microbiome of the organs in search of candidate pathogens; (iv) and to develop sampling and analysis protocols for long‐term, consistent monitoring of the Israeli shoreline. We intend to develop these protocols so they are relevant and applicable to other regions in the world.

## Results

**Figure 2:**
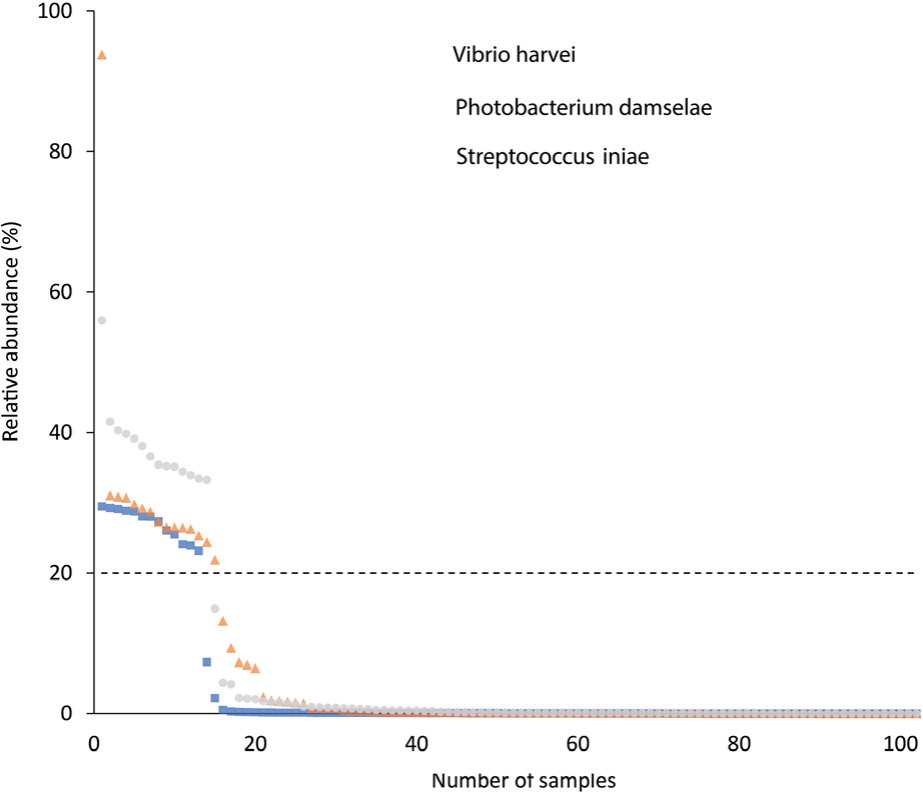
Relative abundance of pathogens in range order across samples. Based on the distribution, we determined a threshold of 20% relative abundance (dash line) for classification of samples positive for *Vibrio harvei*, *Photobacterium damselae* and *Streptococcus iniae* as potential pathogenic bacteria‐infected samples (PPBIS).

All 72 fish from the five fish species were healthy by visual appearance, and no external or internal alterations were observed during necropsy and organ sampling. In total, we obtained 793 759 high‐quality bacterial sequences in 755 OTUs, and these were at the 97% sequence similarity threshold. The three most dominant OTUs were identified as potential pathogens, based on blast against the NCBI database. Two of the OTUs were belonged to the *Vibrionaceae* family: OTU536986 (120 267 sequences, 5.3%) was identified as *P. damselae* and OTU785565 (89 303 sequences, 3.6%) was identified as *V. harveyi*, both were closely related to sequences isolated from diseased Cobia (*Rachycentron canadum*), GenBank accession numbers MH423606 and MH423607 showing 99% and 100% sequences similarity, respectively. In addition, OTU536986 (124 840 sequences, 5.7%) was related to *P. damselae* (MG470847, 99% similarity) which was isolated from Silver pomfret (*Pampus argenteus*) and OTU785565 was related to *V. harveyi* (MK318662, MK318661, 100% identity) which were isolated from tail‐rotted disease of a Whiteleg shrimp (*Litopenaeus vannamei*). The third dominant OTU (OTU4468897) was identified as *S. iniae* and was found to be identical to a genotype isolated from a sick olive flounder (*Paralichthys olivaceus*), CP032401, 100% identity. Relative abundance of the potential pathogens detected was plotted in range order across samples (Fig. 2). Based on the distribution patterns, a threshold of 20% relative abundance for either of the three potential pathogens was determined for classification of samples as PPBIS.

**Figure 3:**
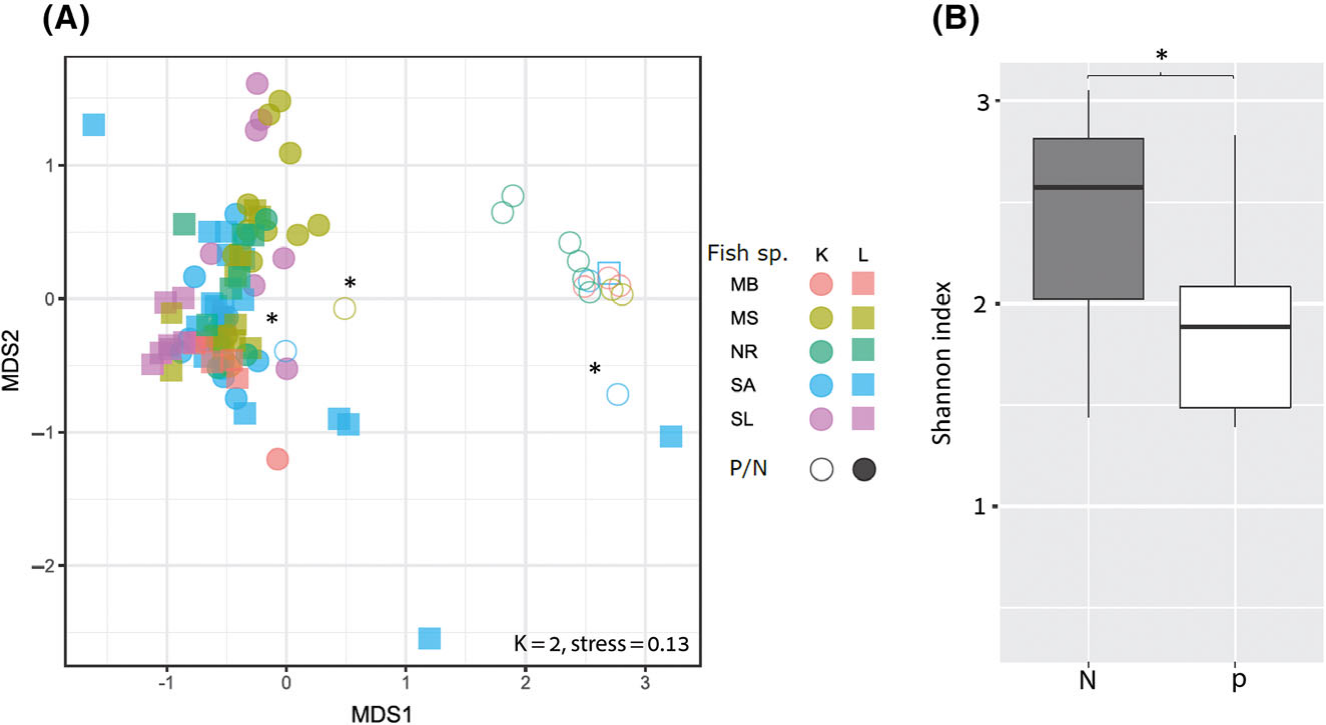
Fish liver and kidney bacterial communities. Microbiome structure was assessed by high‐throughput sequencing of partial 16S rRNA gene fragment. A. Bacterial profiles were compared by non‐metric multidimensional scaling analysis (NMDS) using the Bray–Curtis distance metric. The graph includes samples from four sites (Acre, Kishon, Jaffa and Ashdod), five fish species: *Saurida lessepsianus* (SL), *Sardinella aurita* (SA), *Nemipterus randalli* (NR), *Mullus surmuletus* (MS) and *Mullus barbatus* (MB) and two organs: kidney (K) and liver (L). Each shape and colour represent different fish species and organ (see legend). Empty shapes represent PPBIS (P). B. Box plot of kidney bacterial diversity comparing between PPBIS (P)/non‐PPBIS (N), based on Shannon H’, Kruskal–Wallis chi‐squared = 8.7139, df = 1, *P‐value = 0.003158.

Using these criteria, 16 samples were classified as PPBIS. PPBIS frequency in kidneys was significantly higher (15/16) than in liver samples (Fisher exact test *P* = 0.0008). In total, 23% of all kidney samples were PPBIS. Higher percentages of PPBIS were observed in MB and NR, 75% and 60%, respectively, while no PPBIS were detected in SL. Most of the PPBIS were a combination of the three pathogens and only in three PPBIS, and only one pathogen was above the assigned threshold (*S. iniae* or *P. damselae).* None of the samples contained *V. harveyi* (Table. 1)*.* When comparing the PPBIS according to site area sampling, most of the PPBIS were from fish collected in Acre (31%) and Jaffa (19%). In the site with the highest number of samples, the Kishon, (45/102 samples), only four samples (8%) were identified as PPBIS. No PPBIS were found in fish from Ashdod (Table 2). Non‐metric multidimensional scaling analysis of 102 samples' microbiomes clearly clustered the samples into PPBIS and non‐PPBIS (Fig. 3A). The three PPBIS that contained a single pathogen were clustered apart from their group. We examined differences in bacterial community structure between PPBIS and non‐PPBIS samples by calculating the Shannon index of diversity (Fig. 3B). For this test, only kidney samples were used, as only one PPBIS was obtained from liver samples. Kruskal–Wallis test confirmed higher diversity for non‐PPBIS compared PPBIS kidneys (χ^2^ = 8.71, df = 1, *P* = 0.003). No obvious clustering of samples was noticed based on fish species, site or organ. However, the ADONIS test performed after exclusion of PPBIS samples found significant differences between fish species (*R* ^2^ = 0.11; *P* = 0.001), sites (*R* ^2^ = 0.13; *P* = 0.001) organs (*R* ^2^ = 0.03; *P* = 0.003) and significant species versus site (*R* ^2^ = 0.06; *P* = 0.01) and species versus organ (*R* ^2^ = 0.08; *P* = 0.001) interactions. Nevertheless, the *R* ^2^ values obtained in those comparisons were low, indicating low contribution to variance by these factors.

**Table 1:** Prevalence of potential pathogenic bacteria‐infected samples (PPBIS) in kidney tissue samples.

Potenial pathogen/Species	MB	NR	MS	SA	SL	Total
*Streptococcus iniae*	0	0	1	0	0	1
*Photobacterium damselae*	0	0	0	2	0	2
*Vibrio harveyi*	0	0	0	0	0	0
All 3 pathogens	3	6	2	1	0	12
No. of Kidney samples	4	10	15	16	8	53
PPBIS in Kidney	75%	60%	20%	19%	0%	23%

Prevalence of PPBIS among fish species: *Saurida lessepsian* (SL), *Sardinella aurita *(SA), *Nemipterus randalli* (NR), *Mullus surmuletus* (MS) and *Mullus barbatus* (MB).

**Table 2:** Prevalence of potential pathogenic bacteria‐infected samples (PPBIS) in samples among the four sites.

	Ashdod	Kishon	Yafo	Acre	Total
PPBIS	0	4	7	5	16
No. of samples	5	45	36	16	102
% PPBIS	0	9%	19%	31%	16%

Prevalence of PPBIS, liver and kidney, in all fish sampled in this study according to geographical distribution.

**Figure 4:**
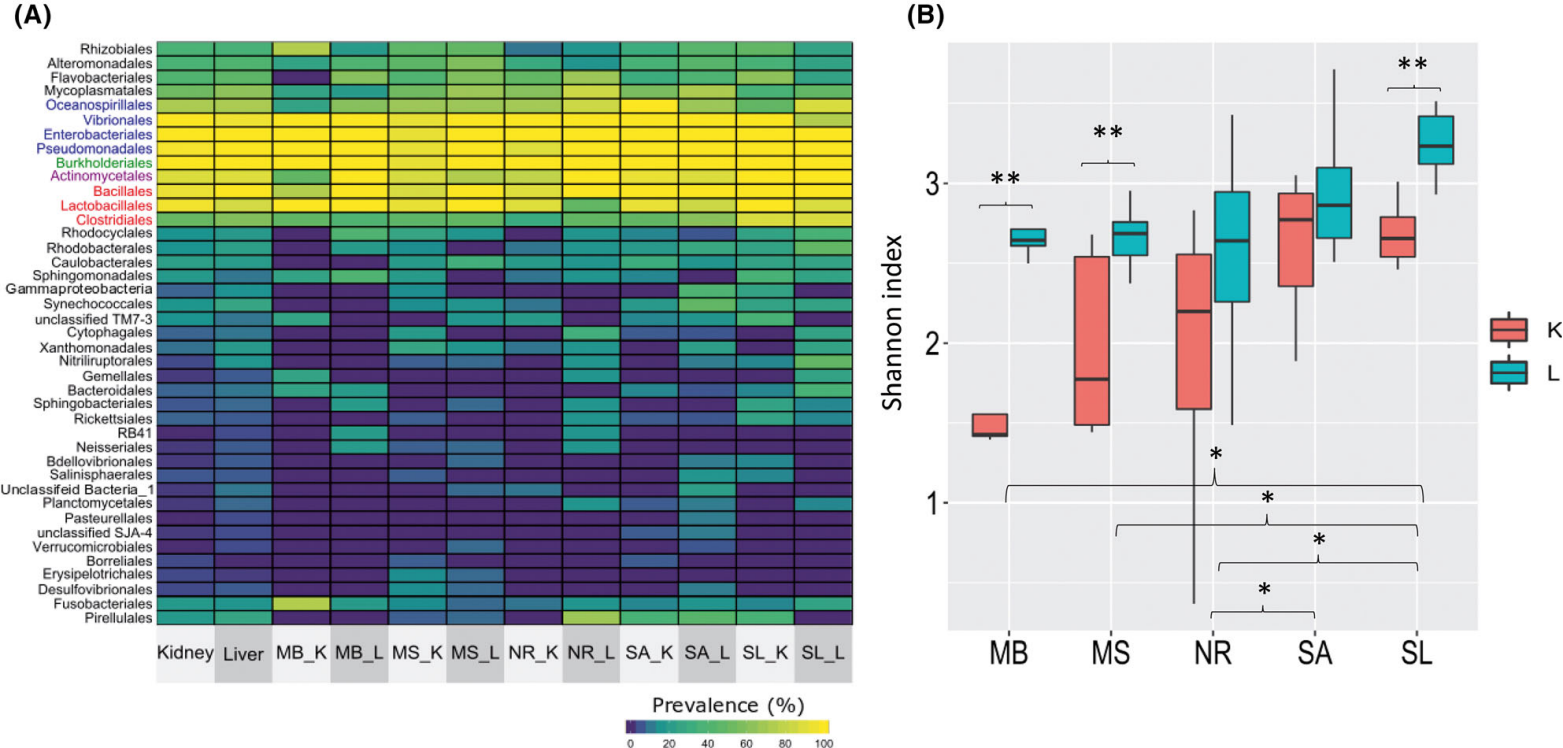
Composition and diversity of fish species and organs bacterial communities. A. Variation in prevalence of bacterial orders in five fish species: *Saurida lessepsianus* (SL), *Sardinella aurita* (SA), *Nemipterus randalli* (NR), *Mullus surmuletus* (MS) and *Mullus barbatus* (MB) and two organs: kidney (K) and liver (L). The heat map includes bacterial orders that appeared at least in two samples. Orders marked by colours are considered core taxa. Blue: Gammaproteobacteria; Green: Betaproteobacteria; Purple: Actinobacteria; Red: Firmicutes. B. Box plot of Shannon index of diversity comparing fish species and organs. Differences between means of organ within species or between species were tested by Kruskal–Wallis test. *P < 0.05; **P < 0.01.

**Figure 5:**
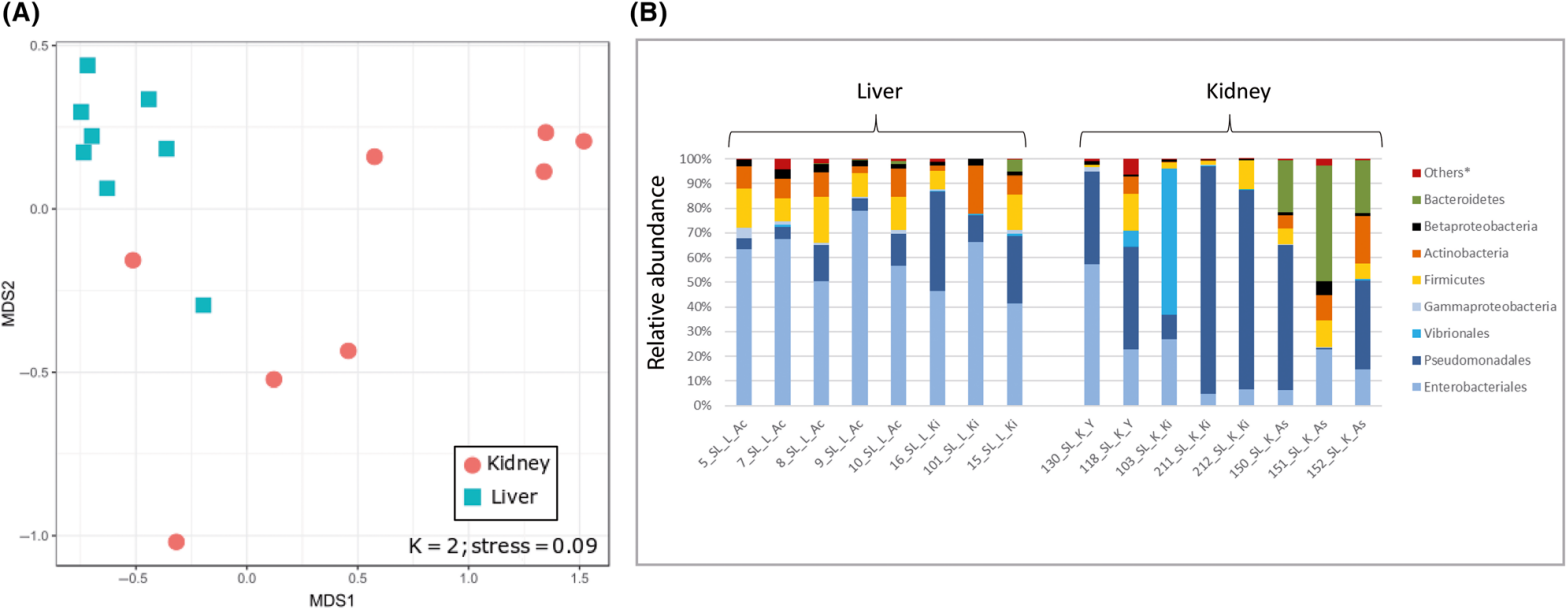
Bacterial communities of *Saurida lessepsianus* kidney and liver samples. A. Bacterial profiles were compared by non‐metric multidimensional scaling analysis (NMDS) using the Bray–Curtis dissimilarity matrix. B. Composition and relative abundance of S. lessepsianus bacterial communities. Site locations: sites: Acre (Ac), Kishon (Ki), Jaffa (Y) and Ashdod (As) All blue shaded groups belonging to Gammaproteobacteria. Others* included all groups under 1% relative abundance.

In light of these results, we questioned whether a core microbiome could be deduced across species and organs. For that, we calculated the prevalence of bacterial taxa at the order level. Indeed, a main core was identified in all samples (Fig. 4A). This core includes four groups belonging to Gammaproteobacteria (Oceanospirillales, Vibrionales, Enterobacteriales and Pseudomonadales), and that three groups which belong to Firmicutes (Lactobacillales, Bacillales and Clostridiales), Betaproteobacteria (Burkholderiales) and Actinobacteria (Actinomycetales). Seven OTUs of Enterobacteriales (Enterobacteriaceae) and one OTU of Burkholderiales appeared in all samples. From Pseudomonadales, 80% of the sequences with high prevalence belonged to *Moraxellaceae* family, with two dominant OTUs: OTU548576 and OTU251317. These OTUs were identical (100%) to bacteria found in the marine environment and marine organisms (MH732572, MH725524, LC184493 and KF881023). Vibrionales and the Lactobacillales appeared in most of the kidney samples (100% and 96%, respectively); these groups included the three pathogens. Analysis of prevalence patterns among fish species and organs pointed to distinct characteristic structures beyond the core (Fig. 4A). We, therefore, compared bacterial diversity (Shannon index) using the factors of species and organs (Fig. 4B). Between species, the overall effect was highly significant (Kruskal–Wallis χ^2^ = 22.2, df = 4, *P* = 0.0002), with SL having highest diversity. Post‐hoc pairwise tests (with Bonferroni corrections) confirmed diversity of SL was higher than all other species but SA. Comparing diversity between liver and kidney samples for each species showed a clear trend of higher diversity for liver compared to kidney samples, with significant values for MB (*P* = 0.014) MS (*P* = 0.0005) and SL (*P* = 0.005). In order to examine the effect of additional parameters (fish species, organ and site) on the microbial community composition, the PPBIS were excluded from the analysis. An ADONIS test showed that each parameter had a significant effect on the microbiota: site (*R* ^2^ = 0.13; *P* = 0.001), fish species (*R* ^2^ = 0.11; *P* = 0.001) and organ (*R* ^2^ = 0.03; *P* = 0.003). An organ effect was demonstrated (Fig. 5) when comparing SL liver and kidney tissues (16 samples in total). For this species, all samples were non‐PPBIS, and thus bias related to this factor was avoided. An ADONIS test significantly separated kidney and liver samples of SL (*R* ^2^ = 0.31; *P* = 0.001). NMDS analysis of these samples (Fig. 5A) demonstrated difference in degree of variance among samples from each organ. Most of the liver samples (7/8) clustered together while the kidney samples were more varied and were only partially clustered according to the Ashdod site. When comparing the bacterial taxonomy in SL tissues (Fig. 5B), Gammaproteobacteria was the most dominant group (74–76%), although in liver tissue the main order was the Enterobacteriales (59%) while in the kidney, Pseudomonadales was the main order (45%). The Vibrionales appear mainly in kidney, mostly within two samples. Actinobacteria and Firmicutes were observed in two tissues (5–11%). Interestingly, a higher percentage of Bacteroidetes appeared only in kidney samples from Ashdod (Fig. 5B).

**Figure 6:**
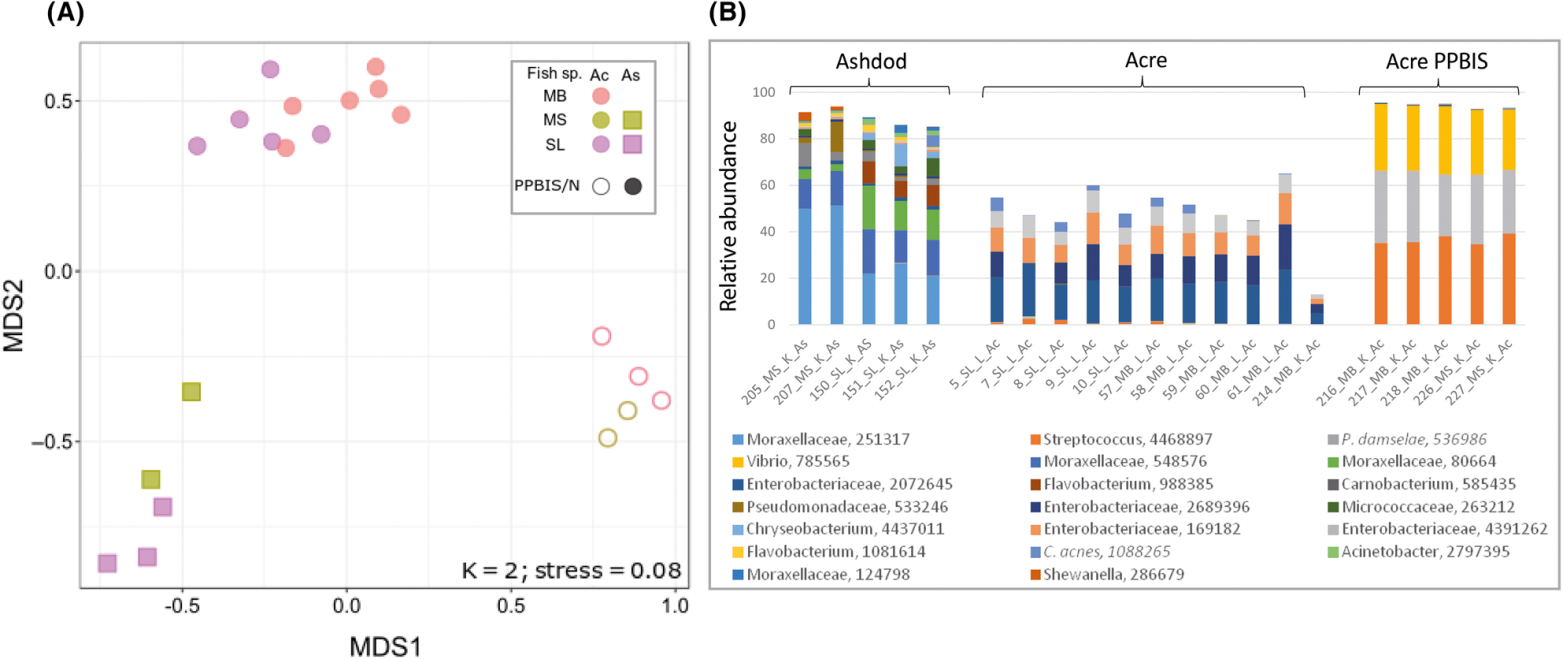
Bacterial communities of fish samples from Acre and Ashdod sites. A. Bacterial profiles of three fish species: *Saurida lessepsianus* (SL*), Mullus surmuletus* (MS) and *Mullus barbatus* (MB) from two sites: Acre (Ac) and Ashdod (As) were compared by non‐metric multidimensional scaling analysis (NMDS) using the Bray–Curtis dissimilarity matrix. B. Composition and relative abundance. The graph represented OTUs with > 0.5% relative abundance.

In order to the effect of site on microbial community, we compared samples collected from Acre and Ashdod sites, the most spatially disparate sites in our study (Fig. 1). These sites included samples from three fish species (MB, AS and MS). Samples from Acre included five PPBIS. We differentiated bacterial community composition between three groups: Ashdod non‐PPBIS, Acre non‐PPBIS and Acre PPBIS. We detected a significant difference (*R* ^2^ = 0.71; *P* = 0.001) and, a pairwise test with Bonferroni correction separated all three groups of samples. NMDS analysis for these samples also confirmed the ADONIS test results (Fig. 6A). NMDS showed evidence of species‐specificity, site and pathogenesis condition. The differences in bacterial community composition and taxonomy are shown in Fig. 6B, based on the dominate OTUs (OTUs that appeared more than 0.5%) at these sites. The first cluster from Ashdod site included three main OTUs that belong to *Moraxellaceae* family (49–68%). At the Acre site, there were no PPBIS. The four main OTUs, belonging to *Enterobacteriaceae* family which contains a single OTU (present in most of the samples) identified as *Cutibacterium acnes* (CP033842 100% similarity), which belongs to Propionibacteriaceae (Actinobacteria). The third cluster, PPBIS from the site (all kidney samples) included mostly the three pathogens (over 92%) as described above (Fig. 6B).

## Discussion

The continental shelf along Israeli shoreline has been monitored for the last few years more extensively due to understanding the importance of studying this region and following temporal ecological changes. This study focused on five common fish species from four sites along the Israeli shoreline and characterized, for the first time, their microbial communities of the kidney and liver. The kidney and liver of 72 fish were sampled and examined to create an initial database. In general, most of fish microbiome studies describe bacteria from skin and intestinal content (Llewellyn *et al.*, 2014). Contrary to previous studies which considered that internal organs which should be sterile in healthy fish (Nieto *et al.*, 1984; Cahill, 1990; Dionne *et al.*, 2009; Salgado‐Miranda *et al.*, 2010), lately, studies have reported, similarly of our study, that bacteria are found also in healthy fish kidneys (Gomez‐Gil, Fajer‐Avila, and García‐Vargas, 2007; Evans and Neff, 2009; Sevellec *et al.*, 2014) and livers (Gomez‐Gil *et al.*, 2007; Salgado‐Miranda *et al.*, 2010). Presence of bacteria within internal organs is still not fully understood and warrants further study. The different results regarding the presence and amount of bacteria in kidney and liver could be explained due to the advances of sensitivity techniques, such as next‐generation sequencing (NGS) and other modern molecular tools. We detected bacteria in 100% of the samples (*n* = 102), which is in contrast to a study that demonstrated bacterial community present in only in 52.6% of freshwater whitefish samples (Sevellec *et al.*, 2014). We suggest that after plotting PPBIS results on graph, a standard of relative abundance needs to be generated. In our case, relative abundance above 20% should dictate the number of samples which have meaningful results regarding the presence of bacteria in fish internal organs. In our study we received, on average, more sequences from fish kidney samples than in the liver (2.5–9 times more), except in SA, were we received less sequences from the kidney tissue, although the liver samples were found to be more diverse in their bacterial community structure (Fig. 4B). No external or internal pathological alterations were observed in the fish; however, screening of their community identified three potential pathogens *Photobacterium damselae, Vibrio harveyi* and *Streptococcus iniae* (99‐100% identity) above 20% relative abundance (Fig. 2) in 16 samples.

*Photobacterium damselae* (not characterized into subspecies level by the sequencing results of this study) is considered a primary pathogen within several species of wild and cultivated marine species such as finfish, elasmobranches, cetaceans and crustaceans (Grimes *et al.*, 1985; Fujioka *et al.*, 1988; Magariños *et al.*, 1992; Fouz *et al.*, 1992; Magariños *et al.*, 1996; Romalde, 2002; Vaseeharan *et al.*, 2007; Labella *et al.*, 2011; Rivas *et al.*, 2013; Valdenegro‐Vega *et al.*, 2013; Costa *et al.*, 2017). *Vibrio harveyi* was reported as a significant pathogen of marine vertebrates and invertebrates (Austin and Zhang, 2006) and can be considered as an opportunistic pathogen, and disease can develop as a result of stress (Chabrillon *et al.*, 2005). *Streptococcus iniae* was responsible for morbidities and mortalities in wild and cultured populations of marine and freshwater fish (Low *et al.*, 1999; Chou *et al.*, 2014) and is considered one of the foremost economically important pathogens in intensive aquaculture (Agnew and Barnes, 2007). In addition, to the ecological importance and potential economic consequences, all three pathogens are known as potentially zoonotic agents (considering *Photobacterium damselae* subsp. *damselae*) and were reported as causing diverse, sometimes severe infections in humans (Weinstein *et al.*, 1997; Asato and Kanaya, 2004; Alvarez *et al.*, 2006). The PPBIS, detected in this study, were clustered together and demonstrated a significantly different bacterial profile clade, compared to all the other samples. In addition, PPBIS diversity (Shannon H’) was significantly lower compared with the rest of the samples. These phenomena, decreased bacterial diversity following disease/stress, are known and described also in humans (Scher *et al.*, 2015; Li *et al.*, 2016). Interestingly, in most of the PPBIS, all the three pathogens were detected together (81% of PPBIS), while *V. harveyi* was not detected alone in any of the samples. Most of the PPBIS were detected in the kidney tissue (only one sample detected PPBIS in the liver). Some pathogens, such as *S. iniae*, are known to have better detection rates in kidney tissue (Bromage *et al.*, 1999; Evans *et al.*, 2001; Agnew and Barnes, 2007). However, in the Türe study (Türe and Alp, 2016), there was no significant difference in the presence of numerous pathogens between the kidney and the liver tissues. Some pathogens have an affinity to specific cells, therefore, for a specific organ, but with marine fish (a group of more than 20 000 different species (Appeltans *et al.*, 2012) with different anatomy and physiology), multi‐organ sampling should be analysed for complete understanding of the fish bacterial community profile. Except for the presence of potential pathogens, we generated a primary database for a bacterial profile that characterizes the kidney and liver microbiome of the five investigated fish species. Nine bacterial groups (taxonomic rank of order) were recognized as the main core of the fish kidney and liver natural microbiota and were common to all fish species. (Fig. 4A). We found that, although the survey included different fish species, organs and geographic locations, there was a group of bacteria that was similar in all of the samples. Vibrionales (Gammaproteobacteria) were one of the main groups that appeared in all kidney samples. Few studies investigated the fish kidney microbiome found different *Vibrio* sp. in healthy kidney blue‐fin tuna (*Thunnus maccoyii*) (Valdenegro‐Vega *et al.*, 2013), spotted rose snapper (*Lutjanus guttatus)* (Gomez‐Gil *et al.*, 2007) and red snapper (*L. campechanus*) (Arias *et al.*, 2013), similar to our results. However, all these studies were based on isolated bacteria contrary to our screen which is based on molecular diagnosis. A limited number of studies have on fish kidney and liver microbiota, however, it would be interesting in the future to compare these results to microbiome results of the same fish species in different marine geographical area and to examine the effect of environmental factors on the bacterial community. We demonstrated significantly different microbial communities of fish internal organs between the Southern (Ashdod) and Northern (Acre) sites. These results may indicate the important influence of the environmental conditions (geography, biotic and abiotic parameter etc.) on the fish microbiome. Compared to terrestrial animals’ epidemiology studies, little research has been done in the marine environment and the few existing studies involved with different fish species, different pathogens and different methods. Therefore, comparing results and understanding trends in this field of marine wild fish microbial epidemiology are challenging. As microbiome studies are becoming popular and the technology of 16S rRna NGS is becoming cheaper, we hope international agencies such as The World Organization for Animal Health (OIE), The World Health Organization (WHO) and The Food and Agriculture Organization (FAO) will support and encourage systemic surveys using this relatively uniform and simple technique. Only a few major global fishing companies fish nearly all the oceans, fishing in more than 50% of the oceans water, and therefore routine systemic sample collection for such surveys is feasible. Due to public health importance, such sample collections might be suggested as a mandatory action in fishing vessels of large global companies. Because epidemiology of wild animals in the marine environment is in its very beginning, it would be wise to strongly establish at this time a constant and uniform diagnostic method so that analysing and comparing of the data will be straightforward, more efficient and better organized.

## Experimental procedures

### Fish and tissue sampling

Wild fish were by trawl fisheries during August–December 2016, from four ports along the Israeli Mediterranean shoreline: Acre, Kishon, Jaffa and Ashdod (Fig. 1). Fish were immediately placed on ice at the boat, transferred to the laboratory, and stored at −20°C until necropsy. It should be mentioned that the fish were obtained at the ports on‐land and at nearby fish markets, so the exact definitive coordinates of the fish capture sites are not acknowledged in this study. All specimens were aseptically dissected for liver and kidney sample collection according to a fish necropsy protocol (Yanong, 2003) with some modifications. The skin area of every fish, where incision was made, was cleaned with ethanol (70% solution) before the fish was dissected. Liver was sampled as soon as it was exposed, and other internal organs were gently removed before sampling the kidney. Necropsy tools were dip‐washed in sodium hypochlorite 6% (Bio‐Lab Ltd., Israel) following cleaning with distillated water between different tissues and different fish. Aseptic sample collection and avoiding cross‐contamination were critical points of this study. All tissue samples were kept frozen at − 80°C until further analysis. Samples of liver (*n* = 49) and kidney (*n* = 53) tissues (102 samples in total) were sub‐sampled from 72 wild marine fish from five different species: *Saurida lessepsianus* (SL), *Sardinella aurita *(SA) , *Nemipterus randalli* (NR), *Mullus surmuletus* (MS) and *Mullus barbatus* (MB) were analysed for their microbial community composition.

### DNA extraction and NGS analyses

DNA was extracted from liver and kidney tissues of each specimen using the Wizard SV Genomic System (Promega, WI, USA) and the genomic DNA purification protocol followed the manufacturer’s instructions for tissue lysates. DNA quantity and purity were estimated using NanoDrop One (NanoDrop Ins., Thermo Scientific), and all isolated genomic DNA was stored at −20°C until use. The template DNA was amplified using GoTaq Green Master Mix (Promega, WI, USA) with T100 thermal cycler (Bio‐Rad, Pleasanton, CA). Partial sequences of the 16S rRNA gene at the V4 hypervariable region were amplified using the following primers (Illumina Tags are in underlined text): CS1‐515F: ACACTGACGACATGGTTCTACA‐GTGCCAGCMGCCGCGGTAA and CS2‐806R: TACGGTAGCAGAGACTTGGTCT‐GGACTACHVGGGTWTCTAAT. The PCR conditions were as follows: denaturation at 94°C for 15 s, annealing at 50°C for 20 s and extension at 72°C for 30 s for 10 cycles with an additional 15 cycles with annealing at 60°C for 20 s. There were 25 cycles in total. PCR products were sent to HyLabs laboratories (Rehovot, Israel) for next‐generation sequencing. Samples were checked by Qubit and Agilent TapeStation, and loaded on the Illumina Miseq, using the V2‐500 cycle kit, sequencing for 2 × 250 bp, which generated paired‐end reads. The data were demultiplexed by Base‐Space, and the FASTQ files were imported to CLC‐bio platform. In CLC‐bio, the reads were trimmed, merged and the subjected to operational taxonomic unit (OTU) analysis in which all reads were trimmed to the same length and OTU's were generated. Then, 16S rRNA taxonomy was determined using the Silva database. CLC‐bio generated the OTU at 97% similarity. Sequencing was performed on 102 tissues samples (a total of 793 759 sequences).

### Statistical analysis

Counts data were normalized using the cumulative sum scaling method (CSS) implemented in the R package ‘metagenomeSeq’ (URL ref. = https://www.nature.com/articles/nmeth.2658). Calculation of the Shannon index of diversity was done in PAST (URL ref. = https://folk.uio.no/ohammer/past/) using rounded normalized counts matrix. The Kruskal–Wallis test was used to compare mean Shannon H’ values between PPBIS positive and negative samples, kidney and livers samples within each species and between fish species. For the later comparison, a Kruskal–Wallis test was followed by post‐hoc Kruskal–Nemenyi test to examine mean differences pair‐wise between species fish species. These tests were conducted in R using the commands: ‘kruskal.test’ and ‘posthoc.kruskal.nemenyi.test’. *P* values were considered significant below 0.05. Non‐metric multidimensional scaling (NMDS) analysis was done based on Bray–Curtis dissimilarities using CSS‐normalized counts with R package ‘vegan’ (version 2.5.3, ref. = https://mran.microsoft.com/snapshot/2015-11-17/web/packages/vegan/vegan.pdf) using the command ‘metaMDS’. NMDS parameters were k = two, try = 100, maxiters = 10 000. Stress values were considered sufficient at values < 0.2. A heat map describing the prevalence of bacterial taxa at the order level was plotted in R using the package ‘superheat’ (version 1.0.0, ref. = https://cran.r-project.org/web/packages/superheat/superheat.pdf). ADONIS tests were used to evaluate the effect of the different factors: fish species, organ and site on bacterial community composition. ADONIS were performed with R package ‘vegan’ using the command ‘adonis’ based on Bray–Curtis dissimilarities with 999 permutations. The resultant *P* values were corrected using the Bonferroni procedure for multiple tests. Where required, post hoc pairwise ADONIS test was conducted R package ‘pairwiseAdonis’ (version 0.0.1, ref. = https://github.com/pmartinezarbizu/pairwiseAdonis) using the command ‘pairwise.adonis’. Boxplots and scatter plots were plotted with R package ‘ggplot2’ (version 3.1.0).

## Conflict of interest

None declared.
